# The League of Mercy

**Published:** 1912-12-21

**Authors:** 


### THE LEAGUE OF MERCY.

### THE ANNUAL MEETING OF PRESIDENTS AT ST. JAMES'S PALACE.

The thirteenth annual meeting of Presidents was held at St. James's Palace on Monday, December 16, His Serene Highness Prince Alexander of Teck, G.C.B., G.C.V.O., D.S.O., Grand President, in the chair.

T.R.H. Princess Alexander of Teck and the Duchess of Albany and their Highnesses Princess Marie Louise and Princess Victoria of Schleswig-Holstein were present. The minutes of the last meeting were read, confirmed, and signed.

### The Grand President's Address.

The Grand President: Your Royal Highness, your Highness, my Lords, Ladies, and Gentlemen,—I would first say that His Majesty the King desires me to assure you of the interest he has taken, and continues to take, in the League, and he trusts that the amount collected will still reach the already excellent sum which it has in the past. His Majesty hopes that it will not be affected so much as had been feared might be the case. This is the first opportunity I have enjoyed of presiding at the annual meeting of Presidents of the League of Mercy, and it gives Princess Alexander and myself great pleasure to be with you on the present occasion. You will, I feel sure, agree with me that it is a great privilege to be permitted to hold this meeting at St. James's Palace, which His Majesty the King has been graciously pleased to place at our disposal. We also much appreciate the admirable arrangements which have been made by the officers of the Lord Chamberlain's Department, and the courtesy they have shown to the officers of the League. During the past year a most successful garden party was given by Mr. and Mrs. Leopold de Rothschild in their beautiful grounds at Gunnersbury Park, when some 2,000 workers for the League had the honour of meeting their Majesties the King and Queen, formerly the Grand Presidents of the League. It has been proposed that a large concert should be given next year, not in aid of the League, but in recognition of the good work done by the members. An influential committee has already been formed to organise and make the necessary arrangements. It is very gratifying to hear that the grants made this year by the League to hospitals outside the area of the distribution of the King's Fund amount to the large sum of £2,305, an increase of £100 over last year's grants.

The Future of Medical Education.

The usefulness of the Samaritan Branch of the League, inaugurated in 1903, whereby workers can secure convalescent home letters, surgical appliances, and other medical and surgical benefits, has been increasingly recognised. It is true that reports have been received from various hospitals and charitable funds which contribute to their support, to the effect that the present year has not been a favourable one in the matter of revenue. The uncertainty surrounding the future of the voluntary hospitals and their honorary staffs is doubtless a factor in the case. Some are apprehensive also as to the future of medical education, which is so closely allied with the work of some of the larger hospitals, and which is already, in part, receiving assistance from public funds. It is true also that under the National Insurance Act power is given to approved societies and to Insurance Committees to subscribe to hospitals, yet the rôle of the voluntary charity in the future, in the provision of medical and surgical benefits for the industrial classes, is not at present clearly evident. The Poor-Law Commissioners' Report reminded us that legislative provision already exists whereby local authorities can establish and maintain hospitals for the treatment of any kind of illness, yet hitherto the voluntary principle has been such a national characteristic that it is only in rare cases that such provision has fallen wholly on the rates in default of any voluntary provision having been made. Under these circumstances, it is a great satisfaction to find that there is hope, I can only say hope, that the total amount for this year will not be less than was collected last year. Since 1899 the League will have paid some £185,000 to the King's Fund, while no less than £12,000 have been distributed by the League among hospitals in the home counties. It is a pleasure to recall the fact that a Vice-President from the Strand district has collected over £50 in pennies, or small subscriptions of less than a shilling each, and a member from Battersea has collected £8 in like amounts. A valuable report was recently made to the King's Fund by Lord Mersey's Committee, which shows that there is little, if any, abuse of the out-patients' departments of the London hospitals by the well-to-do, while methods are suggested whereby ineligible and unsuitable applicants can be properly dealt with. As Grand President and Lady Grand President, the Princess and I take this opportunity of thanking you and all those associated with you for the excellent work you have done on behalf of the League.

Appointment of Officers.

Sir George Alexander, LL.D., J.P., moved: "That the Right Hon. the Lord Farquhar, G.C.V.O., be appointed Chairman; Sir Frederick Green, J.P., Deputy-Chairman; and Capt. A. E. Speer, Hon. Secretary, respectively." The work of these gentlemen in connection with the League was, he said, so well known that no words of his were needed to commend this resolution. Those who had laboured in the same vineyard as their Chairman, Lord Farquhar, knew very well what an excellent agriculturist he was. He endeavoured to sow the seed in likely ground, but he did not leave one there; he watched one until the harvest came home. It was cause for no small congratulation that this year the League would probably be found to have done as well as last year.

Mr. Carl Stettauer, L.C.C., seconded, and it was carried.

Sir Kenneth Matheson, Bart., moved: "That seven representative members of the Executive Committee be appointed: The Right Hon. the Lord Farquhar, G.C.V.O., Colonel H. B. Hamilton, Sir Frederick Green, J.P., Mrs. R. L. Harrison, Mr. Edward Murray Ind, D.L., J.P., Lady Dimsdale, and Sir George Alexander, LL.D., J.P."

Mr. H. W. Keay, J.P., seconded, and it was carried.

The Grand President then presented the Order of Mercy to various ladies and gentlemen who have done zealous work for the League.

### The Treasurer's Statement.

The Hon. Treasurer (Sir Henry Burdett, K.C.B., K.C.V.O.) said he had to offer the meeting very genuine congratulations, because he was in a position to state that, despite all the discouragements and difficulties of the past year, the total result was better than that in 1911. It was not necessary for him to state to the meeting what that meant in work, because he was speaking to those who knew what the difficulties were during 1912. He was sure his hearers were as happy as he himself was proud to know that the result of the year's work had been so satisfactory. In reference to districts, the League had lost three; but they were districts which had never been truly alive; and the League had probably been strengthened by lopping off the dead branches. There were now ninety-one divisions of the League of Mercy, which are going forward vigorously and doing well. And one pleasant feature was that it had got over the stage of very small sums—*i.e.*, less than £10 a year, and everybody now aspired to send something like £100. There were 110 new Vice-Presidents, and that indicated great vitality, inasmuch as it showed there were a large number of persons in a position to influence others to contribute, for each would recruit at least twenty members for the League of Mercy. Against those had to be set seventy-one resignations. The great difficulty in this movement was to keep alive the organisation by filling up the vacancies which occurred every year. He wished he could gather together in a room like this all who had gone off the League, because he thought a little earnest consideration would bring them back again, or the majority. The average yield from each district this year, including all sources, had been £219, as against £214 last year. That showed the utility of special efforts, like the concert at the London Opera House, which had helped the League very much through the South Kensington district. The actual amount received from the districts was £19,912, as against £19,586 last year, an increase of £326.

### A Most Hopeful Year.

He felt this year, as Treasurer, that, when one looked at it squarely as a matter of business, this was one of the most hopeful years in the League's existence. It was a remarkable fact that an increase could be shown in the districts, because there had been many contributing causes, making it difficult for the Grand Presidents, Presidents, Vice-Presidents, and members to keep the League going and get money. His inspiration came from the fact that in a year made bad by circumstances, as 1912 had been, the principle of the League had proved so sound that all concerned had put their shoulders to an extra extent to the work, because they believed in the doctrine of personal service. That, he felt assured, was the underlying cause. The League was the trustee for the small giver. Everyone who worked for the League collected the sixpences and shillings, and by so doing gave a guarantee that every single farthing of such gift would go into the coffers of the hospitals, and be devoted absolutely, without any deduction, to the relief of the sick. Workers had not sat down and said they felt the difficulties were too great and the work was impossible, but, being well themselves, had put vigorous effort forward on behalf of the sick. He, and the officers of the League, had been told that National Insurance was going to make the League of Mercy impossible.

They should not believe a word of it. National Insurance would give everybody connected with the League of Mercy the greatest opportunity they had ever had of being useful to their fellows. There would be an infinite number of difficulties for sick people arising out of National Insurance, and they would require just that help and advice which the Presidents and Vice-Presidents of the League would be able to give. London would thus become a great centre for advice and counsel, which was even more gratifying than the collection of money, for those seriously and dangerously ill. He was not there to speak of the voluntary hospitals at the moment. Their need was great, and their difficulties were great; but he had confidence in the Government that they wanted to have the voluntary character of the hospitals preserved in this country. Those belonging to the League of Mercy could do much to popularise the work, by showing the bearing of the old on the new, and there would arise a hospital system infinitely better and stronger than anything in the past. The League had given away £182,000 to hospitals. This year there was the possibility of being able to give £18,000, and if all did their best there would be the great satisfaction of having reached £200,000. In conclusion, he desired to express the great thanks of the League to the Bishop of London for having come to speak to them, for he had worked and lived in the Metropolis, and was a great encouragement, more especially to its workers. The League was the originator of personal service, although another organisation was using the name. The Bishop would be able to say how much a man could unselfishly render to his fellows, and thus bring happiness to everybody engaged in the work of a great city like this.

### Peace and Mercy at St. James's.

Sir William Job Collins, M.D.: Your Royal Highnesses, my Lords, Ladies, and Gentlemen,—After the opening remarks which have fallen from your Royal Highness and from the Hon. Treasurer, not in the lachrymose vein of last year, but in an optimistic humour, it is unnecessary for any other of the honorary officers to say much. This meeting, I think, will be memorable, at any rate for two things. In the first place, and this is perhaps not the first occasion, Peace and Mercy have met together in adjoining rooms, and I trust that both conferences will draw inspiration and encouragement from the historical associations under which they have met. And, in the second place, this meeting will be memorable because we shall receive the blessing of the Church from the Right Rev. the Lord Bishop of London. He knows very well the intolerable strain of supporting voluntary institutions of another sort. He knows very well the lot of the humbler citizens at any rate. When I first met him, in Bethnal Green, he knew the Barnet Street area, and from that particular area many of those pennies have come to which you, Sir, to-day alluded in the way of small subscriptions. This League is not a Church organisation, but has among its members those belonging to all denominations and to no denomination; it is not a political organisation, for it contains those who belong to both political, or I should say all political, parties, and to no political party.

#### The Value of the Small Worker.

Its work pervades both widely and superficially, and through all classes of society. We have heard of the small worker, and of the small sums sent in, and I heard to-day of a servant girl, now in Montreal, who, out of gratitude to the hospitals of London, has sent over here one guinea per annum to show her gratitude for what was done in those hospitals. You, Sir, and Sir Henry Burdett have alluded to the possibilities of the future. The past year has been characterised by the way in which the small worker has done his part towards the successful issue in the entertainments which were got up by our wealthy supporters. I glean from your remarks, Sir, that the League cannot feel otherwise than somewhat apprehensive as to the future of voluntary hospitals. We know under Section 21 of the Insurance Act that there is power for approved societies to subscribe to the funds of the hospitals. We learnt recently that the medical benefit under the Insurance Act is intended to mean medical benefit given by a general practitioner to private patients, and apparently not to include hospital treatment. If that be so, the need, at any rate in the immediate present, of the work of the voluntary hospitals to continue is self-evident. Going round my wards to-day I took occasion to ascertain how many in my particular wards were insured persons. In a male ward of seventeen, the surgical ward, eight were insured persons. In another of twenty-seven, a medical ward, twelve were insured persons. So it is evident that between 30 and 40 per cent., at any rate in the male wards of our hospitals, will be found to be insured persons, and that work will have to go forward. Where the voluntary hospitals precisely are under the Insurance scheme is not yet clear. The effect of the Insurance scheme upon the management of hospitals and their honorary staffs and their finances, as well as the interest which the charitable public will take in them, is uncertain, and is not yet self-evident, but the immediate need still continues of support to them from the King's Hospital Fund and from this League of Mercy, which is a valuable auxiliary to it. That is not only the best guarantee for this good work, such as we have known it in the past, but is also the best security that it will continue in the future. It is, therefore, my pleasure to say that the League are grateful to receive the encouragement which they have heard to-day to carry on their work. The office work is done with efficiency, and I hope with due economy. They are sorry to lose Mr. Montague Sharpe, who has served for many years, but in his place we have a tried friend, Sir George Alexander. I shall not intervene longer between you and the Bishop of London, who will pronounce the blessing of the Church on the work of the League.

The Right Rev. the Lord Bishop of London then delivered an address, which will be found in another column.*

### Votes of Thanks.

The Rev. W. H. Hornby Steer proposed "That the Right Hon. and Right Rev. the Lord Bishop of London be thanked for his kindness in giving his address." He was sure the address had stirred the hearts of all his hearers, and stimulated them to do even greater things in the future.

Colonel Thomson seconded. He said it was a beautiful picture which the Bishop presented, and his (the speaker's) experience of the East End told him it was also a true one. The Bishop contrasted the man's feelings when he reached the hospital with those he had at home. But what must he feel when he went home again? In many cases the hospital treatment inspired feelings of gratitude in the patient, but in others it had sown the seeds of discontent. Such contrasts caused the birth of anarchism and all that was bad in citizenship. Of course, such feelings added to the difficulties of the work.

The resolution was carried.

The Right Hon. Lord Farquhar, G.C.V.O., moved: "That the Chairman be thanked for his kindness in taking the chair." He thought much of the unexpected success this year was due to the Grand President and Lady Grand President, who were always ready at any moment to help those who were holding a meeting, and to encourage the work of the League. And her Highness Princess Victoria had been one of the chief supports of the League ever since its inauguration. He must also mention the Duchess of Albany and Princess Victoria of Schleswig-Holstein, and the League was most grateful to them.

Sir Frederick Green, J.P., seconded, emphasising the great help which the members of the Royal Family had been, but for whose splendid example and encouragement the enthusiasm in some quarters would not have been so pronounced.

The meeting then terminated.

[The amount of the total collection from each district will be published in The Hospital early in the New Year as soon as all the contributions have been received. To publish the totals now would not do full justice to those districts which have not yet been able to get in all the money.]

* Members of the League may like to know that this Address will be republished in an attractive form, and copies may be obtained from any bookseller or from The Scientific Press, 28 and 29 Southampton Street, Strand, London, W.C.

